# Gender-related risk factors for surgical site infections. Results from 10 years of surveillance in Germany

**DOI:** 10.1186/s13756-019-0547-x

**Published:** 2019-06-03

**Authors:** Seven Johannes Sam Aghdassi, Christin Schröder, Petra Gastmeier

**Affiliations:** 1 Charité – Universitätsmedizin Berlin, corporate member of Freie Universität Berlin, Humboldt-Universität zu Berlin, and Berlin Institute of Health, Institute of Hygiene and Environmental Medicine, Berlin, Germany; 2 National Reference Center for Surveillance of Nosocomial Infections, Berlin, Germany

**Keywords:** Gender, Surgical site infection, Surveillance, Infection control, Healthcare-associated infection

## Abstract

**Background:**

Surgical site infections (SSI) are among the most frequently occurring healthcare-associated infections worldwide. Various analyses to determine risk factors have been conducted in the past, generally attributing a higher SSI-risk to male patients. However, when focusing on specific procedures, this is not always true. Our objective was to identify for which procedures male or female sex represents an independent risk factor for SSI and which parameters may explain these differences.

**Methods:**

We used the database of surgical procedures from the German national nosocomial infection surveillance system. We included procedures conducted between 2008 and 2017. We excluded procedures solely executed for one sex (e.g. mastectomy) and procedures with 20,000 or fewer operations. The observed outcome was the occurrence of SSI. All models were adjusted for confounders, which were eliminated with backward selection. The following factors were included in the analysis: age, ASA score, wound contamination class, duration of surgery, and season. All models contained the investigated factor sex.

**Results:**

Sixteen procedure types with 1,266,782 individual procedures and 18,824 SSI were included. Overall, the incidence rate ratio and the adjusted odds ratio for SSI were significantly higher for male patients. The included individual procedures were grouped into five surgical categories. For orthopedics and traumatology as well as abdominal surgery, SSI-rates were significantly higher for male patients. For heart and vascular surgery, SSI-rates were significantly higher for female patients. Other included surgical categories and individual procedures yielded diverse results. Similar results were found when solely analyzing deep and organ-space SSI. Multivariable analysis for attributable gender-related risk factors revealed differences with regard to underlying risk factors.

**Conclusions:**

SSI-rates differ by sex for certain procedures. When examining underlying risk factors, differences between male and female patients can be demonstrated. Our analysis considered a limited number of parameters, which were not sufficient to fully explain the observed differences. Further studies are required to obtain a more comprehensive understanding of the topic and to include gender-specific aspects into future SSI-prevention strategies.

**Electronic supplementary material:**

The online version of this article (10.1186/s13756-019-0547-x) contains supplementary material, which is available to authorized users.

## Background

Surgical site infections (SSI; *abbreviation used for singular and plural meaning of the term*) are among the most frequently occurring healthcare associated infections [1, 2]. A patient’s risk to acquire a SSI is influenced by a broad range of factors. Besides procedure-related risk factors, patient-related risk factors have to be recognized. Sex is a factor, which plays an important role when estimating the probability of disease and complication across all fields of medicine. With regard to SSI, previous studies have demonstrated that generally, SSI occur more frequently in men than in women [3–5]. However, when looking at specific procedure types, this statement is not always accurate. For instance, when comparing SSI-rates in men and women for certain types of heart surgery, SSI occur more frequently in female patients [6, 7].

The large data base of surgical procedures and SSI available at the German national nosocomial infection surveillance system (Krankenhaus-Infektions-Surveillance-System; KISS) provides the opportunity to investigate the differences in SSI-rates among men and women systematically for a large number of procedure types. As an additional objective, we investigated whether underlying risk factors for SSI differ between male and female patients, in order to explain existing gender-related differences.

## Methods

The definitions and methods of the SSI-component of KISS (OP-KISS) have been described earlier [8, 9]. In general, KISS is using the methodology of the United States National Healthcare Surveillance Network (NHSN) for surveillance of SSI [10]. In accordance with the NHSN methodology, the data collected in OP-KISS include age and sex, as well as the risk factors belonging to the so-called “National Nosocomial Infections Surveillance System (NNIS) risk index”. These are: duration of surgery, wound contamination class (WCC) and American Society of Anesthesiologists (ASA) score. For every procedure these parameters were recorded. Another parameter that we added to our analyses was season, which, as it has been previously described, has an impact on SSI-rates for several procedure types [11].

Age was not viewed as a continuous variable but instead grouped into four age groups. Duration of operation was considered as categories based on the quartiles per procedure type. ASA scores were categorized as defined by the American Society of Anesthesiologists [12]. WCC was segregated into clean and clean-contaminated (i.e. 1 and 2), and contaminated and dirty-infected (i.e. 3 and 4).

Data utilized for all analyses were from a 10-year surveillance period from 2008 to 2017. SSI were considered for analysis when they occurred within 30 days or 90 days after the procedure depending on the procedure type (90 days for procedures with an implant).

To increase the robustness of data, the following criteria were applied:

• Procedures were removed from the analyses if an individual surgical department provided less than 30 procedures for a specific procedure type.

• Only procedure types with more than 20,000 procedures were included in the final analyses.

• Procedures types only performed in men or women, such as prostatectomy, mastectomy, and hysterectomy, were also excluded.

Generalized linear models were applied to estimate the association between the occurrence of SSI and the available factors. Since observations within an operative department are not statistically independent, generalized estimating equation models were calculated to account for this clustering effect. For the outcome SSI following an operation, a binomial distribution in the generalized estimating equation model was applied.

The selected outcome was occurrence of SSI. All models were adjusted for confounders. First, all confounders were included in the model and then eliminated from the full model with backward selection (*p*-value > 0.05). The following variables were considered: age (35–54, 55–74, ≥ 75 vs. < 35), ASA score (≤ 2 vs. > 2), WCC (≤ 2 vs. > 2), duration (≤ quartile 1, > quartile 1 & ≤ quartile 2, > quartile 2 & ≤ quartile 3 vs. > quartile 3), and season (spring (March, April, May), summer (June, July, August), autumn (September, October, November) vs. winter (December, January, February)). All models contained the investigated factor sex. Adjusted odds ratios with 95% confidence intervals for the occurrence of SSI were calculated. A variable was considered as a significant risk factor if the lower limit of the 95% confidence interval was greater than 1, and as a significant protective factor if the upper limit of the 95% confidence interval was less than 1.

Analyses were conducted for SSI in general, as well as separately for deep incisional and organ-space SSI. All analyses were performed using R 3.4.3 (R Foundation for Statistical Computing, Vienna, Austria) and SAS 9.4 (SAS Institute Inc., Cary, NC, USA).

For the purpose of this article, the terms “sex” and “gender” are used synonymously.

### Ethical approval

All data were anonymized and collected in accordance to paragraph 23 of the German federal law, German Protection against Infection Act (“*Infektionsschutzgesetz*”), which regulates the prevention and control of infectious diseases in humans. Therefore, ethical approval and informed consent were not required.

## Results

Following the above stated inclusion criteria, 16 different procedure types were included in the analyses. We grouped all included procedures into five surgical categories, representing different surgical subspecialties. These were:

• four procedures from the field of orthopedics and traumatology (hip prosthesis following arthrosis and fracture, knee prosthesis, and arthroscopic procedures),

• four procedures from the field of abdominal surgery (endoscopic cholecystectomy, endoscopic and open colon surgery, and endoscopic appendectomy),

• four procedures from the field of heart and vascular surgery (coronary artery bypass grafting (CABG) (separated into procedures including vein harvesting and procedures without vein harvesting), re-vascularization of arterial occlusion, and venous stripping),

• one neurosurgical procedure (lumbar disk surgery),

• and three surgical procedures from the field of general surgery (endoscopic and open hernia repair, and thyroid surgery).

Altogether, 1,266,728 procedures were included in the analyses, 587,253 performed in men and 679,529 in women. Table a, which can be found in Additional file 1 summarizes the demographics of the included patients, as well as data on other parameters included in our analyses.

Overall, for all procedures included, incidence rates were significantly higher for male patients than for female patients. This ratio remained unchanged after adjusting for the selected risk factors. When comparing infection rates between male and female patients for the five listed surgical categories, we can see that male sex represented a significant risk factor for the group of orthopedics and traumatology, as well as abdominal surgery. Conversely, female sex was identified as a significant risk factor for heart and vascular surgery and general surgery (as defined by the individual procedures included listed above). For neurosurgery, SSI-rates did not differ significantly by sex.

**Fig. 1:**
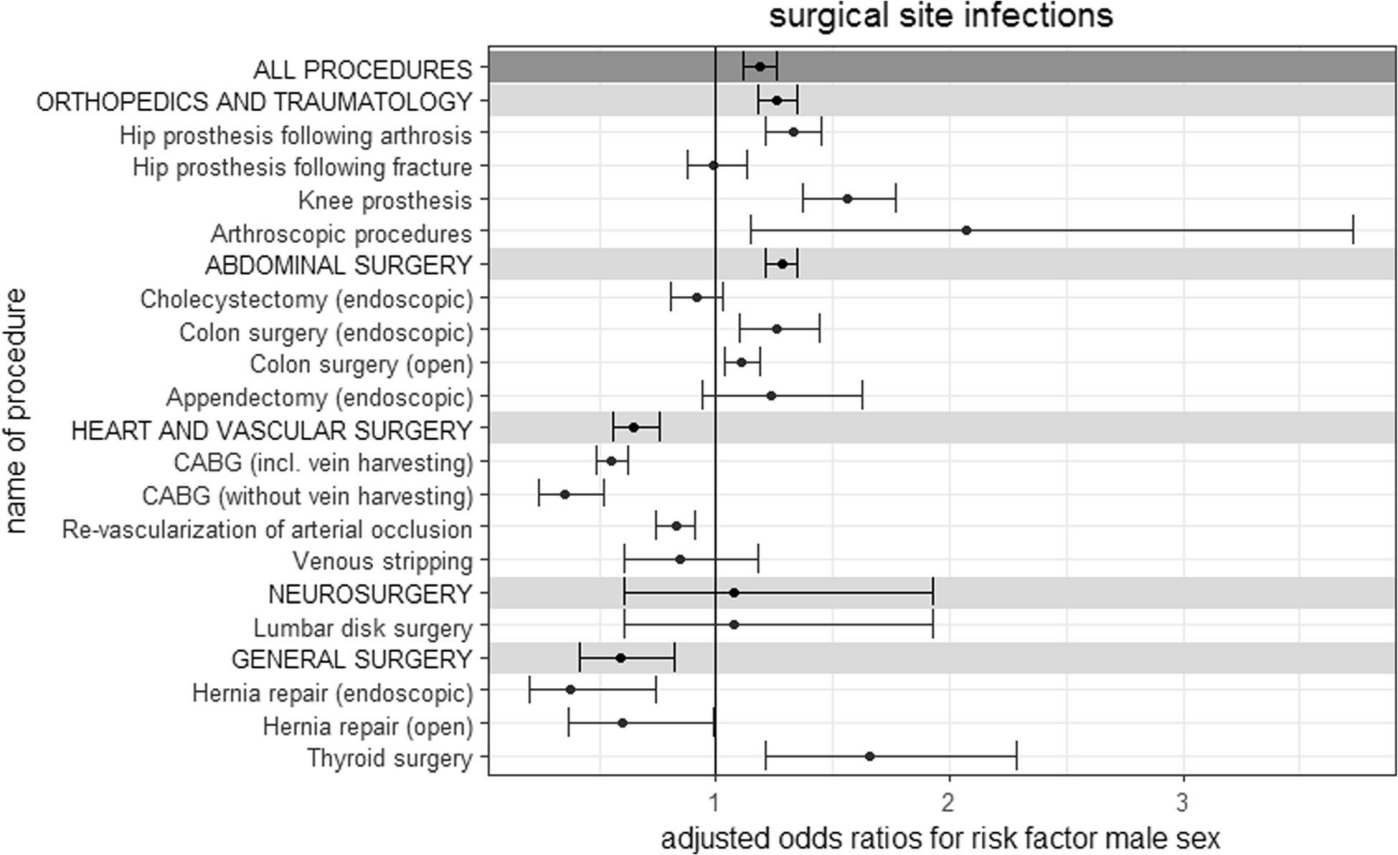
Adjusted odds ratios for the risk factor male sex for the occurrence of surgical site infections. *CABG* coronary artery bypass grafting

When distinguishing further between the respective procedures, male sex was a significant risk factor for all individual orthopedic and trauma procedures except hip prosthesis following fracture, for which no significant differences were identified between female and male patients. Abdominal surgery procedures yielded results that were more diverse. Male sex represented a significant risk factor for colon surgery (endoscopic and open), whereas no significant differences between sexes were found for endoscopic cholecystectomy and endoscopic appendectomy. All individual procedures subsumed under the category heart and vascular surgery, except venous stripping, identified female sex as a significant risk factor. For the category, labelled general surgery, different results were found by procedure type. For both endoscopic and open hernia repair, SSI-rates were significantly higher in female patients. For thyroid surgery, the opposite was true. These results are further illustrated in Table 1 and Fig. 1.

**Table 1:** Surgical site infection rates per surgical category and procedure type adjusted for the risk factor male sex

	Female sex			Male sex			IRR (95% CI)	AOR (95% CI)
	N	SSI	%	N	SSI	%		
All procedures	679,529	8847	1.30	587,253	9977	1.70	1.31 (1.27–1.34)	1.19 (1.12–1.26)
Orthopedics and traumatology	448,287	3077	0.69	287,290	2390	0.83	1.21 (1.15–1.28)	1.26 (1.18–1.35)
Hip prosthesis following arthrosis	222,430	1476	0.66	155,513	1357	0.87	1.32 (1.22–1.42)	1.33 (1.21–1.45)
Hip prosthesis following fracture	53,480	995	1.86	22,801	437	1.92	1.03 (0.92–1.15)	0.99 (0.88–1.13)
Knee prosthesis	159,102	591	0.37	92,223	550	0.60	1.61 (1.43–1.81)	1.56 (1.37–1.77)
Arthroscopic procedures	13,275	15	0.11	16,753	46	0.27	2.43 (1.36–4.46)	2.07 (1.15–3.73)
Abdominal surgery	138,543	3653	2.64	94,426	3687	3.90	1.50 (1.43–1.57)	1.28 (1.21–1.35)
Cholecystectomy (endoscopic)	83,623	651	0.78	44,552	374	0.84	1.08 (0.95–1.23)	0.92 (0.81–1.03)
Colon surgery (endoscopic)	13,193	565	4.28	10,786	594	5.51	1.29 (1.15–1.44)	1.26 (1.10–1.44)
Colon surgery (open)	27,864	2368	8.50	27,455	2641	9.62	1.15 (1.08–1.21)	1.11 (1.04–1.19)
Appendectomy (endoscopic)	13,863	69	0.50	11,633	78	0.67	1.35 (0.97–1.86)	1.24 (0.94–1.63)
Heart and vascular surgery	52,060	1958	3.76	124,457	3660	2.94	0.78 (0.73–0.82)	0.65 (0.56–0.76)
CABG (incl. Vein harvesting)	18,209	1022	5.61	70,014	2250	3.21	0.56 (0.52–0.60)	0.55 (0.49–0.62)
CABG (without vein harvesting)	4627	328	7.09	18,803	503	2.68	0.36 (0.31–0.41)	0.35 (0.24–0.52)
Re-vascularization of arterial occlusion	14,263	523	3.67	27,579	868	3.15	0.85 (0.77–0.95)	0.83 (0.74–0.91)
Venous stripping	14,961	85	0.57	8061	39	0.48	0.85 (0.58–1.24)	0.85 (0.61–1.18)
Neurosurgery	16,103	59	0.37	19,127	75	0.39	1.07 (0.76–1.51)	1.08 (0.61–1.93)
Lumbar disk surgery	16,103	59	0.37	19,127	75	0.39	1.07 (0.76–1.51)	1.08 (0.61–1.93)
General surgery	24,536	100	0.41	61,953	165	0.27	0.65 (0.51–0.84)	0.59 (0.42–0.82)
Hernia repair (endoscopic)	3679	9	0.24	31,725	32	0.10	0.41 (0.20–0.86)	0.38 (0.20–0.74)
Hernia repair (open)	3871	19	04.9	23,868	85	0.36	0.72 (0.44–1.19)	0.60 (0.37–0.99)
Thyroid surgery	16,986	72	0.42	6360	48	0.75	1.79 (1.24–2.57)	1.66 (1.21–2.29)

*N* number, *SSI* Surgical site infection(s), *IRR* incidence rate ratio, *AOR* adjusted odds ratio, *CI* Confidence interval, *CABG* Coronary artery bypass grafting

Analyses were also conducted separately for deep and organ-space SSI. As in the group of all SSI, the overall incidence rate ratio for all included procedures was significantly higher for male patients. After adjusting for the included risk factors, the adjusted odds ratio was also significantly higher for male patients. When looking at the results for deep and organ-space SSI for the five surgical categories listed above, the same differences with regard to adjusted odds ratios were observed as for SSI of all depths. Unlike for SSI of all depths, the adjusted odds ratio for deep and organ-space SSI was significantly higher for male patients undergoing endoscopic cholecystectomy. Moreover, unlike for SSI of all depths, for deep and organ-space SSI no statistically significant differences between sexes were found for re-vascularization of arterial occlusion, as well as endoscopic and open hernia repair. However, these procedures still showed a higher incidence rate of deep and organ-space SSI in female patients. Additional information can be found in Table 2.

**Table 2:** Deep and organ-space surgical site infection rates per surgical category and procedure type adjusted for the risk factor male sex

	Female sex			Male sex			IRR(95% CI)	AOR (95% CI)
	N	SSI	%	N	SSI	%		
All procedures	679,529	5295	0.78	587,253	6360	1.08	1.39 (1.34–1.45)	1.27 (1.18–1.36)
Orthopedics and traumatology	448,287	2327	0.52	287,290	1946	0.68	1.31 (1.23–1.39)	1.36 (1.26–1.47)
Hip prosthesis following arthrosis	222,430	1099	0.49	155,513	1103	0.71	1.44 (1.32–1.56)	1.46 (1.32–1.60)
Hip prosthesis following fracture	53,480	803	1.50	22,801	354	1.55	1.04 (0.91–1.17)	0.99 (0.87–1.14)
Knee prosthesis	159,102	411	0.26	92,223	448	0.49	1.89 (1.65–2.16)	1.83 (1.59–2.11)
Arthroscopic procedures	13,275	14	0.11	16,753	41	0.24	2.32 (1.27–4.26)	1.96 (1.10–3.47)
Abdominal surgery	138,543	1717	1.24	94,426	2030	2.15	1.75 (1.64–1.87)	1.48 (1.37–1.60)
Cholecystectomy (endoscopic)	83,623	224	0.27	44,552	199	0.45	1.67 (1.38–2.02)	1.26 (1.02–1.55)
Colon (endoscopic)	13,193	293	2.22	10,786	361	3.35	1.53 (1.31–1.78)	1.47 (1.25–1.72)
Colon (open)	27,864	1163	4.17	27,455	1434	5.22	1.27 (1.17–1.37)	1.23 (1.12–1.34)
Appendectomy (endoscopic)	13,863	37	0.27	11,633	36	0.31	1.16 (0.73–1.84)	1.24 (0.94–1.63)
Heart and vascular surgery	52,060	1174	2.26	124,457	2281	1.83	0.81 (0.75–0.87)	0.68 (0.56–0.82)
CABG (incl. Vein harvesting)	18,209	690	3.79	70,014	1511	2.16	0.56 (0.51–0.61)	0.55 (0.48–0.64)
CABG (without vein harvesting)	4627	205	4.43	18,803	300	1.60	0.35 (0.29–0.42)	0.35 (0.22–0.56)
Re-vascularization of arterial occlusion	14,263	261	1.83	27,579	458	1.66	0.91 (0.78–1.06)	0.87 (0.75–1.02)
Venous stripping	14,961	18	0.12	8061	12	0.15	1.24 (0.60–2.57)	1.24 (0.67–2.30)
Neurosurgery	16,103	35	0.22	19,127	43	0.22	1.03 (0.66–1.62)	1.03 (0.44–2.45)
Lumbar disk surgery	16,103	35	0.22	19,127	43	0.22	1.03 (0.66–1.62)	1.03 (0.44–2.45)
General surgery	24,536	42	0.17	61,953	60	0.10	0.57 (0.38–0.84)	0.51 (0.33–0.79)
Hernia repair (endoscopic)	3679	3	0.08	31,725	13	0.04	0.50 (0.14–1.76)	0.50 (0.14–1.80)
Hernia repair (open)	3871	9	0.23	23,868	27	0.11	0.49 (0.23–1.04)	0.48 (0.23–1.01)
Thyroid surgery	16,986	30	0.18	6360	20	0.31	1.78 (1.01–3.14)	1.62 (1.09–2.39)

*N* number, *SSI* surgical site infection(s), *IRR* Incidence rate ratio, *AOR* adjusted odds ratio, *CI* confidence interval, *CABG* coronary artery bypass grafting

A multivariable analysis was conducted to investigate whether risk factors for SSI differ between male and female patients. When comparing results for the five surgical categories, we observed that for all categories, except orthopedics and traumatology, differences in the underlying significant gender-related risk factors existed. Correspondingly, for deep and organ-space SSI, significant differences were found for three out of five surgical categories, namely, abdominal surgery, neurosurgery, and general surgery. Unlike for all SSI, no significant differences in the underlying gender-related risk factors were revealed for heart and vascular surgery. On the level of individual procedures, differences in the underlying significant risk factors were found in a total of 11 procedures. When analyzed for deep and organ-space separately, significant differences in risk factors were revealed for 11 procedures as well. The results of the multivariable analyses are illustrated in table b and table c to be found in Additional file 2.

## Discussion

We analyzed over one million surgical procedures performed in a timespan of a decade to investigate the influence of sex on SSI. Evidently, gender-related differences existed not only with regard to the occurrence of SSI, but also in reference to underlying risk factors. As it had been demonstrated before, men were generally at a higher risk of SSI [3, 7]. Our analyses, which distinguished between all SSI, as well as deep and organ-space SSI separately, was able to reveal that this applied also to deeper SSI which generally pose a more severe complication. When examining different surgical categories, we were able to demonstrate that SSI-rates were significantly higher in men undergoing orthopedic and trauma procedures. A prevalence study investigating predictors of colonization with *Staphylococcus species* in patients undergoing cardiac and orthopedic surgery found significantly higher colonization rates in men [13]. With *Staphylococcus aureus* being the most common SSI-causing pathogen, it is conceivable that this disparity in colonization patterns could partially explain the gender-related differences in SSI-rates. When looking at specific procedures in the group of orthopedics and traumatology we can conclude that for hip prosthesis following fracture the risk for SSI did not differ significantly by sex. This result corresponded with the findings of Gibbons et al. in their systematic review published in 2011 [14]. Interestingly, we found differences in the underlying risk factors for SSI after hip prosthesis following fracture between female and male patients, indicating that a higher ASA score, higher WCC, and longer duration of surgery had a bigger effect on female than male patients.

For abdominal surgery, male patients were at a significantly higher risk for SSI. Similar results have been demonstrated for a variety of abdominal surgical procedures. For instance, Mazmudar et al. found male sex to be a significant risk factor for adverse outcomes in general, and specifically for SSI in patients undergoing elective pancreatectomy [5]. Similarly, Warren et al. demonstrated that male sex is an independent risk factor for SSI following cholecystectomy [4]. Unlike the results of Warren’s study, we identified male patients to only have a significantly higher risk for deep and organ-space SSI than female patients when undergoing cholecystectomy. For SSI of all depths, this finding was not repeated in our data. However, following our inclusion and exclusion criteria, we only included laparoscopic cholecystectomies, whereas Warren et al. included open cholecystectomies as well. When discussing their findings, Warren and coauthors refer to the fact that the male patients they included had a more severe biliary tract disease and thereby a higher likelihood of undergoing open cholecystectomy. Open cholecystectomy is generally associated with a higher SSI-rate than a laparoscopic approach [15, 16].

In contrast to our results, a retrospective case-control study published by Pedroso-Fernandez et al. attributed a significantly higher risk for SSI to female patients undergoing colorectal surgery [17]. However, it has to be noted that with a total of 911 included patients the study had a rather low power. We included a much higher number of cases in our analyses and were able to demonstrate a significantly higher risk for SSI in male patients undergoing colon surgery.

The adjusted odds ratios for SSI in female patients undergoing heart and vascular surgery were significantly higher than in male patients. This is primarily due to the higher SSI-rates after CABG and re-vascularization of arterial occlusion. Other studies have yielded similar results [6, 18, 19]. While our analyses confirmed these findings, the underlying reasons are not fully clear. Some authors have argued in the past that differences in fat distribution between men and women may explain these differences, with women typically having more fat tissue in their hips and thighs in case of re-vascularization of arterial occlusion [18]. It seems conceivable that the same may apply to sternal wound infections. Studies have indicated that obesity is a significant risk factor for sternal SSI following CABG [19]. The presence of more fat tissue in the chest region in female patients, therefore, appears to provide a possible explanation. Another possible reason for the higher SSI-rate in women following CABG, could be the fact that these patients, generally, have more severe comorbidities than their male counterparts [20]. However, Koch et al. argue that after adjusting for comorbidities, sex does not pose an independent risk factor for a worse outcome with regard to survival. Since our analyses and previous studies only included limited information on comorbidity (i.e. in our case the NNIS-risk index), the higher SSI-rates in women may simply be attributable to an overall worse condition. A study by Si and coauthors did not find significant differences in the SSI-risk of women and men undergoing CABG [21].

Our data revealed that the SSI-risk for female patients undergoing hernia repair was significantly higher than for male patients. This result is consistent with previously published data [22]. For thyroid surgery, male sex was identified to correlate with a significantly higher SSI-risk. To our best knowledge, this represents a new finding and has not been described previously for this procedure type.

Our study had several strengths and limitations. The most relevant strengths were the high number of included procedures and SSI, as well as the long duration of observation. This, in combination with the adjustment for various risk factors, enabled us to investigate the impact of the factor sex on the outcome SSI with high accuracy. Due to the large number of departments participating in OP-KISS, our data provides, to a certain extent, a basis for extrapolations to a national level. Limitations of our analyses existed due to the methodology applied in KISS, which is based upon voluntary participation and data transfer to the National Reference Center for Surveillance of Nosocomial Infections. Here, the heterogeneity of data collectors of the participating departments has to be recognized. Furthermore, OP-KISS is a patient-based surveillance module in which patients should be observed for the occurrence of SSI even after discharge from the hospital. The quality of data gathered from this “post-discharge surveillance” differs greatly between the participating departments. Following the KISS-methodology, only a limited amount of patient- and procedure-related data were collected. Detailed data concerning comorbidities, as well as underlying immunomodifying factors were not available and could not be included in the analyses. Additionally, it has to be noted that differences in bacterial skin colonization significantly correlate with the parameter sex [23]. The impact of this effect on our data remains unknown.

Despite these limitations, our data provides solid information on the interaction of sex and the occurrence of SSI. In the future, the data could be utilized for establishing a “tailor-made”, patient-oriented, and individual approach to the prevention of SSI and infection prevention and control in general. This could for instance mean that in the future, certain preoperative decolonizing regimes might differ by sex or other preventive measures could be executed for female or male patients only.

## Conclusion

To our best knowledge, our analyses represent the most comprehensive analyses to this date that investigated SSI-occurrence in relation to sex. Our analyses revealed diverse results, which on one hand reinforced previous studies linking male sex to higher SSI-rates, and on the other hand demonstrated that gender-related SSI-rates differ greatly by procedure type. Our multivariable analysis to identify underlying risk factors to explain these gender-related differences, did not reveal a clear pattern, which one could use to infer detailed information on “female- or male-only” risk factors from. Further studies will be needed to investigate the influence of sex on SSI-occurrence and to identify gender-specific risk factors. When this is done, an emphasis should be placed on differences in male and female microbiome composition, as well as underlying comorbidities.

## Additional files

Additional file 1: **Table S1.** Description of dataset and included variables. (DOCX 42 kb)

Additional file 2: **Table S2.** Multivariable analysis of risk factors for the occurrence of surgical site infections separated for female and male patients. (DOCX 127 kb)

## Data Availability

The datasets used and analyzed in the context of this survey are available from the corresponding author upon reasonable request.

## Abbreviations

ASA: American Society of Anesthesiologists
CABG: coronary artery bypass grafting
KISS: Krankenhaus-Infektions-Surveillance-System
NHSN: National Healthcare Surveillance Network
NNIS: National Nosocomial Infections Surveillance System
OP-KISS: SSI-component of KISS
SSI: surgical site infection(s)
WCC: wound contamination class
