# Supplementary material for: Gender-related risk factors for surgical site infections. Results from 10 years of surveillance in Germany
Source: Antimicrob Resist Infect Control. 2019 Jun 3;8:95. doi: 10.1186/s13756-019-0547-x (PMC6547551; doi:10.1186/s13756-019-0547-x)
Supplement: Supplementary file 1 — Table S1. Description of dataset and included variables. (DOCX 42 kb) [file 13756_2019_547_MOESM1_ESM.docx]

**Table S1** Description of dataset and included variables

| **Surgical category or individual procedure** | **Sex** | **Procedures**  **N**  **(%)** | **ASA**  **>2**  **N**  **(%)** | **Median age in years (Q1, Q3)** | **WCC >2**  **N**  **(%)** | **Spring**  **N**  **(%)** | **Summer**  **N**  **(%)** | **Autumn**  **N**  **(%)** | **Winter**  **N**  **(%)** | **Median duration of surgery (min)**  **(Q1, Q3)** |
| --- | --- | --- | --- | --- | --- | --- | --- | --- | --- | --- |
| **All procedures** | All | 1266782 (100) | 490428 (38.7) | 68  (57, 76) | 85279 (6.7) | 327712 (25.9) | 291119 (23.0) | 340885  (26.9) | 307066 (24.2) | 76  (56, 108) |
|  | F | 679529 (53.6) | 236984 (34.9) | 70  (59, 77) | 45199 (6.7) | 178993 (26.3) | 156519 (23.0) | 183633  (27.0) | 160384 (23.6) | 73  (55, 97) |
|  | M | 587253 (46.4) | 253444 (43.2) | 67  (56, 75) | 40080 (6.8) | 148719 (25.3) | 134600 (22.9) | 157252  (26.8) | 146682 (25.0) | 82  (59, 125) |
| **Orthopedics and traumatology** | All | 735577  (100) | 259873 (35.3) | 71  (62, 78) | 2659  (0.4) | 191389 (26.0) | 163419 (22.2) | 205055  (27.9) | 175714 (23.9) | 73  (58, 91) |
|  | F | 448287 (60.9) | 160505 (35.8) | 73  (64, 79) | 1509  (0.3) | 119161 (26.6) | 101622 (22.7) | 123359  (27.5) | 104145 (23.2) | 73  (58, 90) |
|  | M | 287290 (39.1) | 99368 (34.6) | 69  (60, 76) | 1150  (0.4) | 72228 (25.1) | 61797 (21.5) | 81696  (28.4) | 71569  (24.9) | 75  (58, 93) |
| Hip prosthesis following arthrosis | All | 377943  (100) | 118519 (31.4) | 70  (62, 77) | 809  (0.2) | 99055 (26.2) | 84273 (22.3) | 106024  (28.1) | 88591  (23.4) | 70  (55, 88) |
|  | F | 222430 (58.9) | 68777 (30.9) | 72  (64, 78) | 458  (0.2) | 59532 (26.8) | 50813 (22.8) | 61718  (27.7) | 50367  (22.6) | 70  (55, 87) |
|  | M | 155513 (41.1) | 49742 (32.0) | 68  (59, 75) | 351  (0.2) | 39523 (25.4) | 33460 (21.5) | 44306  (28.5) | 38224  (24.6) | 71  (56, 90) |
| Hip prosthesis following fracture | All | 76281  (100) | 54713 (71.7) | 82  (76, 88) | 945  (1.2) | 19138 (25.1) | 18727 (24.6) | 19082  (25.0) | 19334  (25.3) | 72  (58, 90) |
|  | F | 53480  (70.1) | 37792 (70.7) | 83  (77, 88) | 650  (1.2) | 13457 (25.2) | 13215 (24.7) | 13383  (25.0) | 13425  (25.1) | 71  (57, 89) |
|  | M | 22801  (29.9) | 16921 (74.2) | 80  (73, 86) | 295  (1.3) | 5681 (24.9) | 5512 (24.2) | 5699  (25.0) | 5909  (25.9) | 74  (60, 92) |
| Knee prosthesis | All | 251325  (100) | 83635 (33.3) | 71  (63, 76) | 345  (0.1) | 65288 (26.0) | 53223 (21.2) | 72425  (28.8) | 60389  (24.0) | 80  (65, 99) |
|  | F | 159102 (63.3) | 52406 (32.9) | 71  (63, 77) | 194  (0.1) | 42470 (26.7) | 34450 (21.7) | 45049  (28.3) | 37133  (23.3) | 80  (65, 97) |
|  | M | 92223  (36.7) | 31229 (33.9) | 69  (61, 76) | 151  (0.2) | 22818 (24.7) | 18773 (20.4) | 27376  (29.7) | 23256  (25.2) | 84  (68, 102) |
| Arthroscopic procedures | All | 30028  (100) | 3006 (10.0) | 49  (32, 61) | 560  (1.9) | 7908 (26.3) | 7196 (24.0) | 7524  (25.1) | 7400  (24.6) | 35  (21, 57) |
|  | F | 13275  (44.2) | 1530 (11.5) | 53  (40, 65) | 207  (1.6) | 3702 (27.9) | 3144 (23.7) | 3209  (24.2) | 3220  (24.3) | 30  (20, 50) |
|  | M | 16753  (55.8) | 1476 (8.8) | 45  (28, 58) | 353  (2.1) | 4206 (25.1) | 4052 (24.2) | 4315  (25.8) | 4180  (25.0) | 36  (23, 61) |
| **Abdominal surgery** | All | 232969  (100) | 65982 (28.3) | 59  (44, 73) | 81108 (34.8) | 59823 (25.7) | 57707 (24.8) | 59298  (25.5) | 56141  (24.1) | 71  (47, 116) |
|  | F | 138543 (59.5) | 34633 (25.0) | 58  (42, 72) | 43111 (31.1) | 35836 (25.9) | 34226 (24.7) | 35332  (25.5) | 33149  (23.9) | 65  (45, 105) |
|  | M | 94426  (40.5) | 31349 (33.2) | 61  (47, 73) | 37997 (40.2) | 23987 (25.4) | 23481 (24.9) | 23966  (25.4) | 22992  (24.3) | 80  (51, 130) |
| Cholecystectomy (endoscopic) | All | 128175  (100) | 26464 (20.6) | 56  (43, 70) | 32808 (25.6) | 33162 (25.9) | 30811 (24.0) | 32657  (25.5) | 31545  (24.6) | 58  (43, 78) |
|  | F | 83623  (65.2) | 14620 (17.5) | 54  (41, 68) | 18075 (21.6) | 21799 (26.1) | 20060 (24.0) | 21329  (25.5) | 20435  (24.4) | 55  (42, 75) |
|  | M | 44552  (34.8) | 11844 (26.6) | 60  (47, 72) | 14733 (33.1) | 11363 (25.5) | 10751 (24.1) | 11328  (25.4) | 11110  (24.9) | 62  (46, 83) |
| Colon surgery (endoscopic) | All | 23979  (100) | 7084 (29.5) | 63  (53, 74) | 10375 (43.3) | 6024 (25.1) | 5962 (24.9) | 6389  (26.6) | 5604  (23.4) | 138  (106, 180) |
|  | F | 13193  (55.0) | 3829 (29.0) | 65  (55, 74) | 5791 (43.9) | 3327 (25.2) | 3323 (25.2) | 3548  (26.9) | 2995  (22.7) | 134  (104, 174) |
|  | M | 10786  (45.0) | 3255 (30.2) | 62  (51, 73) | 4584 (42.5) | 2697 (25.0) | 2639 (24.5) | 2841  (26.3) | 2609  (24.2) | 142  (110, 185) |
| Colon surgery (open) | All | 55319  (100) | 31069 (56.2) | 72  (60, 79) | 22711 (41.1) | 14297 (25.8) | 14386 (26.0) | 13708  (24.8) | 12928 (23.4) | 132  (100, 174) |
|  | F | 27864  (50.4) | 15579 (55.9) | 73  (62, 81) | 11507 (41.3) | 7224 (25.9) | 7322 (26.3) | 6891  (24.7) | 6427  (23.1) | 128  (97, 169) |
|  | M | 27455  (49.6) | 15490 (56.4) | 70  (59, 78) | 11204 (40.8) | 7073 (25.8) | 7064 (25.7) | 6817  (24.8) | 6501  (23.7) | 136  (104, 179) |
| Appendectomy (endoscopic) | All | 25496  (100) | 1365 (5.4) | 28  (20, 45) | 15214 (59.7) | 6340 (24.9) | 6548 (25.7) | 6544  (25.7) | 6064  (23.8) | 40  (31, 54) |
|  | F | 13863  (54.4) | 605 (4.4) | 26  (19, 43) | 7738 (55.8) | 3486 (25.1) | 3521 (25.4) | 3564  (25.7) | 3292  (23.7) | 40  (30, 53) |
|  | M | 11633  (45.6) | 760 (6.5) | 30  (20, 47) | 7476 (64.3) | 2854 (24.5) | 3027 (26.0) | 2980  (25.6) | 2772  (23.8) | 41  (32, 55) |
| **Heart and vascular surgery** | All | 176517  (100) | 142672 (80.8) | 69  (60, 75) | 1009  (0.6) | 45503 (25.8) | 41266 (23.4) | 45463  (25.8) | 44285  (25.1) | 165  (114, 210) |
|  | F | 52060  (29.5) | 35594 (68.4) | 70  (60, 77) | 340  (0.7) | 13616 (26.2) | 11145 (21.4) | 14116  (27.1) | 13183  (25.3) | 131  (63, 187) |
|  | M | 124457 (70.5) | 107078 (86.0) | 68  (60, 75) | 669  (0.5) | 31887 (25.6) | 30121 (24.2) | 31347  (25.2) | 31102  (25.0) | 175  (133, 218) |
| CABG (incl. vein harvesting) | All | 88223  (100) | 84975 (96.3) | 70  (62, 76) | 49  (0.1) | 22748 (25.8) | 21717 (24.6) | 21871  (24.8) | 21887  (24.8) | 187  (156, 226) |
|  | F | 18209  (20.6) | 17579 (96.5) | 73  (66, 77) | 12  (0.1) | 4764 (26.2) | 4498 (24.7) | 4607  (25.3) | 4340  (23.8) | 182  (151, 220) |
|  | M | 70014  (79.4) | 67396 (96.3) | 69  (61, 75) | 37  (0.1) | 17984 (25.7) | 17219 (24.6) | 17264  (24.7) | 17547  (25.1) | 190  (158, 228) |
| CABG (without vein harvesting) | All | 23430  (100) | 21749 (92.8) | 68  (59, 75) | 8  (0.0) | 6042  (25.8) | 5810  (24.8) | 5919  (25.3) | 5659  (24.2) | 179  (145, 216) |
|  | F | 4627  (19.7) | 4333 (93.6) | 71  (63, 77) | 0  (0.0) | 1224 (26.5) | 1107 (23.9) | 1165 (25.2) | 1131 (24.4) | 166  (135, 206) |
|  | M | 18803  (80.3) | 17416 (92.6) | 67  (59, 74) | 8  (0.0) | 4818 (25.6) | 4703 (25.0) | 4754 (25.3) | 4528 (24.1) | 180  (150, 219) |
| Re- vascularization of arterial occlusion | All | 41842  (100) | 32224 (77.0) | 71  (63, 78) | 776  (1.9) | 10612 (25.4) | 10729 (25.6) | 10593  (25.3) | 9908  (23.7) | 130  (90, 188) |
|  | F | 14263  (34.1) | 11339 (79.5) | 75  (67, 82) | 231  (1.6) | 3596 (25.2) | 3665 (25.7) | 3603  (25.3) | 3399  (23.8) | 120  (80, 175) |
|  | M | 27579  (65.9) | 20885 (75.7) | 69  (62, 76) | 545  (2.0) | 7016 (25.4) | 7064 (25.6) | 6990  (25.3) | 6509  (23.6) | 136  (94, 193) |
| Venous stripping | All | 23022  (100) | 3724 (16.2) | 56  (46, 67) | 176  (0.8) | 6101 (26.5) | 3010 (13.1) | 7080  (30.8) | 6831  (29.7) | 50  (40, 69) |
|  | F | 14961  (65.0) | 2343  (15.7) | 56  (46, 67) | 97  (0.6) | 4032 (27.0) | 1875 (12.5) | 4741  (31.7) | 4313  (28.8) | 50  (38, 65) |
|  | M | 8061  (35.0) | 1381 (17.1) | 56  (46, 66) | 79  (1.0) | 2069 (25.7) | 1135 (14.1) | 2339  (29.0) | 2518  (31.2) | 55  (40, 70) |
| **Neurosurgery** | All | 35230  (100) | 5739 (16.3) | 54  (44, 67) | 59  (0.2) | 8349 (23.7) | 8939 (25.4) | 9558  (27.1) | 8384  (23.8) | 70  (50, 100) |
|  | F | 16103  (45.7) | 2633 (16.4) | 55  (44, 69) | 31  (0.2) | 3828 (23.8) | 4097 (25.4) | 4406  (27.4) | 3772  (23.4) | 70  (50, 101) |
|  | M | 19127  (54.3) | 3106 (16.2) | 54  (44, 66) | 28  (0.1) | 4521 (23.6) | 4842 (25.3) | 5152  (26.9) | 4612  (24.1) | 70  (50, 100) |
| Lumbar disk surgery | All | 35230  (100) | 5739 (16.3) | 54  (44, 67) | 59  (0.2) | 8349 (23.7) | 8939 (25.4) | 9558  (27.1) | 8384  (23.8) | 70  (50, 100) |
|  | F | 16103  (45.7) | 2633 (16.4) | 55  (44, 69) | 31  (0.2) | 3828 (23.8) | 4097 (25.4) | 4406  (27.4) | 3772  (23.4) | 70  (50, 101) |
|  | M | 19127  (54.3) | 3106 (16.2) | 54  (44, 66) | 28  (0.1) | 4521 (23.6) | 4842 (25.3) | 5152  (26.9) | 4612  (24.1) | 70  (50, 100) |
| **General surgery** | All | 86489  (100) | 16162 (18.7) | 59  (47, 71) | 444  (0.5) | 22648 (26.2) | 19788 (22.9) | 21511  (24.9) | 22542  (26.1) | 63  (45, 91) |
|  | F | 24536  (28.4) | 3619 (14.7) | 55  (44, 68) | 208  (0.8) | 6552 (26.7) | 5429 (22.1) | 6420  (26.2) | 6135  (25.0) | 80  (54, 114) |
|  | M | 61953  (71.6) | 12543 (20.2) | 60  (48, 72) | 236  (0.4) | 16096 (26.0) | 14359 (23.2) | 15091  (24.4) | 16407  (26.5) | 59  (43, 81) |
| Hernia repair (endoscopic) | All | 35404  (100) | 5143 (14.5) | 58  (47, 69) | 76  (0.2) | 9134 (25.8) | 8245 (23.3) | 8779  (24.8) | 9246  (26.1) | 55  (40, 75) |
|  | F | 3679  (10.4) | 647 (17.6) | 60  (45, 74) | 20  (0.5) | 975  (26.5) | 866  (23.5) | 945  (25.7) | 893  (24.3) | 52  (38, 70) |
|  | M | 31725  (89.6) | 4496 (14.2) | 58  (47, 69) | 56  (0.2) | 8159 (25.7) | 7379 (23.3) | 7834  (24.7) | 8353  (26.3) | 55  (40, 76) |
| Hernia repair (open) | All | 27739  (100) | 8216 (29.6) | 66  (52, 76) | 211  (0.8) | 7258 (26.2) | 6622 (23.9) | 6586  (23.7) | 7273  (26.2) | 55  (40, 71) |
|  | F | 3871  (14.0) | 1130 (29.2) | 68  (46, 78) | 55  (1.4) | 1031 (26.6) | 965  (24.9) | 933  (24.1) | 942  (24.3) | 45  (33, 60) |
|  | M | 23868  (86.0) | 7086 (29.7) | 66  (52, 75) | 156  (0.7) | 6227 (26.1) | 5657 (23.7) | 5653  (23.7) | 6331  (26.5) | 55  (42, 73) |
| Thyroid surgery | All | 23346  (100) | 2803 (12.0) | 54  (45, 64) | 157  (0.7) | 6256 (26.8) | 4921 (21.1) | 6146  (26.3) | 6023  (25.8) | 100  (75, 132) |
|  | F | 16986  (72.8) | 1842 (10.8) | 53  (44, 63) | 133  (0.8) | 4546 (26.8) | 3598 (21.2) | 4542  (26.7) | 4300  (25.3) | 96  (72, 127) |
|  | M | 6360  (27.2) | 961 (15.1) | 56  (48, 65) | 24  (0.4) | 1710 (26.9) | 1323 (20.8) | 1604  (25.2) | 1723  (27.1) | 111  (83, 145) |

*N* number; *ASA* American Society of Anesthesiologists; *WCC* wound contamination class; *Q1* first quartile; *Q3* third quartile; *min* minutes; *F* female; *M* male; *CABG* coronary artery bypass grafting
