# Supplementary material for: Gender-related risk factors for surgical site infections. Results from 10 years of surveillance in Germany
Source: Antimicrob Resist Infect Control. 2019 Jun 3;8:95. doi: 10.1186/s13756-019-0547-x (PMC6547551; doi:10.1186/s13756-019-0547-x)
Supplement: Supplementary file 2 — Table S2. Multivariable analysis of risk factors for the occurrence of surgical site infections separated for female and male patients. (DOCX 127 kb) [file 13756_2019_547_MOESM2_ESM.docx]

**Table S2** Multivariable analysis of risk factors for the occurrence of surgical site infections separated for female and male patients

| **Surgical category** | **Procedure** | **Variable** | **Group** | **OR women**  (95% CI) | **OR men**  (95% CI) |
| --- | --- | --- | --- | --- | --- |
| **All** |  | Age | <35 | reference | reference |
|  |  |  | 35-54 | 1.64 (1.29-2.08) | 1.67 (1.40-1.99) |
|  |  |  | 55-74 | 1.90 (1.47-2.44) | 2.04 (1.70-2.45) |
|  |  |  | ≥ 75 | 1.98 (1.51-2.59) | 2.17 (1.79-2.63) |
|  |  | ASA | >2 | reference | reference |
|  |  |  | 1-2 | 0.32 (0.29-0.36) | 0.38 (0.34-0.42) |
|  |  | WCC | >2 | reference | reference |
|  |  |  | 1-2 | 0.26 (0.22-0.33) | 0.32 (0.27-0-37) |
|  |  | Duration of surgery | Q4 | reference | reference |
|  |  |  | Q1 | 0.57 (0.50-0.64) | 0.59 (0.53-0.66) |
|  |  |  | Q2 | 0.64 (0.58-0.71) | 0.71 (0.66-0.78) |
|  |  |  | Q3 | 0.78 (0.73-0.84) | 0.83 (0.78-0.89) |
|  |  | Season | Winter | reference | reference |
|  |  |  | Spring | 1.06 (0.99-1.13) | 1.01 (0.95-1.07) |
|  |  |  | Summer | 1.04 (0.98-1.11) | 1.14 (1.07-1.21) |
|  |  |  | Autumn | 1.18 (1.11-1.26) | 0.99 (0.93-1.05) |
| **Orthopedics and traumatology** | **All** | Age | <35 | reference | reference |
|  |  |  | 35-54 | 3.65 (1.50-8.93) | 1.57 (1.03-2.39) |
|  |  |  | 55-74 | 4.13 (1.73-9.90) | 1.77 (1.15-2.72) |
|  |  |  | ≥ 75 | 6.06 (2.54-14.46) | 2.38 (1.54-3.68) |
|  |  | ASA | >2 | reference | reference |
|  |  |  | 1-2 | 0.40 (0.37-0.44) | 0.55 (0.49-0.61) |
|  |  | WCC | >2 | reference | reference |
|  |  |  | 1-2 | 0.28 (0.16-0.50) | 0.27 (0.15-0.45) |
|  |  | Duration of surgery | Q4 | reference | reference |
|  |  |  | Q1 | 0.45 (0.39-0.52) | 0.53 (0.46-0.63) |
|  |  |  | Q2 | 0.57 (0.51-0.64) | 0.67 (0.58-0.76) |
|  |  |  | Q3 | 0.72 (0.66-0.80) | 0.81 (0.72-0.91) |
|  |  | Season | Winter | reference | reference |
|  |  |  | Spring | 1.10 (0.99-1.21) | 1.00 (0.87-1.15) |
|  |  |  | Summer | 1.32 (1.18-1.47) | 1.18 (1.05-1.34) |
|  |  |  | Autumn | 1.07 (0.96-1.18) | 1.06 (0.93-1.20) |
|  | Hip prosthesis following arthrosis | Age | <35 | reference | reference |
|  |  |  | 35-54 | 4.17 (0.58-29.73) | 1.21 (0.50-2.90) |
|  |  |  | 55-74 | 5.58 (0.80-38.89) | 1.51 (0.63-3.61) |
|  |  |  | ≥ 75 | 6.18 (0.88-43.30) | 1.81 (0.76-4.33) |
|  |  | ASA | >2 | reference | reference |
|  |  |  | 1-2 | 0.40 (0.35-0.46) | 0.55 (0.48-0.63) |
|  |  | WCC | >2 | reference | reference |
|  |  |  | 1-2 | 0.28 (0.12-0.63) | 0.29 (0.16-0.54) |
|  |  | Duration of surgery | Q4 | reference | reference |
|  |  |  | Q1 | 0.39 (0.32-0.48) | 0.49 (0.40-0.60) |
|  |  |  | Q2 | 0.55 (0.48-0.64) | 0.70 (0.59-0.83) |
|  |  |  | Q3 | 0.69 (0.60-0.79) | 0.79 (0.68-0.92) |
|  |  | Season | Winter | reference | reference |
|  |  |  | Spring | 1.18 (1.02-1.37) | a |
|  |  |  | Summer | 1.40 (1.20-1.63) | a |
|  |  |  | Autumn | 1.10 (0.94-1.28) | a |
|  | Hip prosthesis following fracture | Age | <35 | reference¹ | reference¹ |
|  |  |  | 35-54 |  |  |
|  |  |  | 55-74 | a | a |
|  |  |  | ≥ 75 | a | a |
|  |  | ASA | >2 | reference | reference |
|  |  |  | 1-2 | 0.56 (0.48-0.66) | a |
|  |  | WCC | >2 | reference | reference |
|  |  |  | 1-2 | 0.49 (0.23-1.04) | a |
|  |  | Duration of surgery | Q4 | reference | reference |
|  |  |  | Q1 | 0.56 (0.45-0.70) | a |
|  |  |  | Q2 | 0.68 (0.57-0.81) | a |
|  |  |  | Q3 | 0.78 (0.66-0.92) | a |
|  |  | Season | Winter | reference | reference |
|  |  |  | Spring | a | a |
|  |  |  | Summer | a | a |
|  |  |  | Autumn | a | a |
|  | Knee prosthesis | Age | <35 | reference¹ | reference¹ |
|  |  |  | 35-54 |  |  |
|  |  |  | 55-74 | a | 0.74 (0.56-0.97) |
|  |  |  | ≥ 75 | a | 1.04 (0.76-1.42) |
|  |  | ASA | >2 | reference | reference |
|  |  |  | 1-2 | 0.55 (0.45-0.67) | 0.65 (0.52-0.81) |
|  |  | WCC | >2 | reference | reference |
|  |  |  | 1-2 | 0.14 (0.07-0.30) | 0.11 (0.05-0.24) |
|  |  | Duration of surgery | Q4 | reference | reference |
|  |  |  | Q1 | 0.44 (0.34-0.57) | 0.59 (0.45-0.76) |
|  |  |  | Q2 | 0.50 (0.39-0.65) | 0.59 (0.47-0.73) |
|  |  |  | Q3 | 0.75 (0.60-0.94) | 0.80 (0.65-0.98) |
|  |  | Season | Winter | reference | reference |
|  |  |  | Spring | 1.23 (1.00-1.51) | 1.33 (1.01-1.75) |
|  |  |  | Summer | 1.47 (1.17-1.85) | 1.61 (1.12-2.08) |
|  |  |  | Autumn | 1.08 (0.84-1.40) | 1.39 (1.10-1.77) |
|  | Arthroscopic procedures | Age | <35 | reference | reference¹ |
|  |  |  | 35-54 | a |  |
|  |  |  | 55-74 | a | a |
|  |  |  | ≥ 75 | a | a |
|  |  | ASA | >2 | reference | reference |
|  |  |  | 1-2 | a | a |
|  |  | WCC | >2 | reference | reference |
|  |  |  | 1-2 | a | a |
|  |  | Duration of surgery | Q4 | reference | reference |
|  |  |  | Q1 | a | 0.11 (0.03-0.35) |
|  |  |  | Q2 | a | 0.31 (0.14-0.71) |
|  |  |  | Q3 | a | 0.70 (0.33-1.51) |
|  |  | Season | Winter | reference | reference |
|  |  |  | Spring | a | a |
|  |  |  | Summer | a | a |
|  |  |  | Autumn | a | a |
| **Abdominal surgery** | All | Age | <35 | reference | reference |
|  |  |  | 35-54 | 1.73 (1.34-2.24) | 1.89 (1.54-2.33) |
|  |  |  | 55-74 | 3.25 (2.54-4.16) | 3.06 (2.46-3.80) |
|  |  |  | ≥ 75 | 3.92 (2.96-5.19) | 3.29 (2.60-4.17) |
|  |  | ASA | >2 | reference | reference |
|  |  |  | 1-2 | 0.44 (0.39-0.48) | 0.49 (0.44-0.54) |
|  |  | WCC | >2 | reference | reference |
|  |  |  | 1-2 | 0.65 (0.56-0.75) | a |
|  |  | Duration of surgery | Q4 | reference | reference |
|  |  |  | Q1 | 0.62 (0.53-0.73) | 0.66 (0.55-0.80) |
|  |  |  | Q2 | 0.65 (0.58-0.74) | 0.76 (0.67-0.86) |
|  |  |  | Q3 | 0.77 (0.70-0.85) | 0.83 (0.75-0.92) |
|  |  | Season | Winter | reference | reference |
|  |  |  | Spring | a | a |
|  |  |  | Summer | a | a |
|  |  |  | Autumn | a | a |
|  | Cholecystectomy (endoscopic) | Age | <35 | reference | reference |
|  |  |  | 35-54 | 0.79 (0.57-1.08) | 0.67 (0.39-1.14) |
|  |  |  | 55-74 | 1.34 (1.06-1.70) | 1.52 (0.93-2.47) |
|  |  |  | ≥ 75 | 1.85 (1.33-2.59) | 2.25 (1.30-3.90) |
|  |  | ASA | >2 | reference | reference |
|  |  |  | 1-2 | 0.68 (0.56-0.82) | 0.62 (0.50-0.79) |
|  |  | WCC | >2 | reference | reference |
|  |  |  | 1-2 | a | a |
|  |  | Duration of surgery | Q4 | reference | reference |
|  |  |  | Q1 | 0.61 (0.46-0.80) | 0.48 (0.34-0.68) |
|  |  |  | Q2 | 0.61 (0.47-0.79) | 0.56 (0.42-0.76) |
|  |  |  | Q3 | 0.78 (0.63-0.97) | 0.63 (0.48-0.82) |
|  |  | Season | Winter | reference | reference |
|  |  |  | Spring | a | a |
|  |  |  | Summer | a | a |
|  |  |  | Autumn | a | a |
|  | Colon surgery (endoscopic) | Age | <35 | reference | reference |
|  |  |  | 35-54 | a | a |
|  |  |  | 55-74 | a | a |
|  |  |  | ≥ 75 | a | a |
|  |  | ASA | >2 | reference | reference |
|  |  |  | 1-2 | 0.68 (0.55-0.85) | 0.64 (0.53-0.76) |
|  |  | WCC | >2 | reference | reference |
|  |  |  | 1-2 | a | a |
|  |  | Duration of surgery | Q4 | reference | reference |
|  |  |  | Q1 | 0.55 (0.40-0.75) | 0.61 (0.44-0.86) |
|  |  |  | Q2 | 0.55 (0.42-0.74) | 0.89 (0.69-1.15) |
|  |  |  | Q3 | 0.65 (0.49-0.86) | 0.89 (0.71-1.12) |
|  |  | Season | Winter | reference | reference |
|  |  |  | Spring | a | a |
|  |  |  | Summer | a | a |
|  |  |  | Autumn | a | a |
|  | Colon surgery (open) | Age | <35 | reference | reference |
|  |  |  | 35-54 | a | a |
|  |  |  | 55-74 | a | a |
|  |  |  | ≥ 75 | a | a |
|  |  | ASA | >2 | reference | reference |
|  |  |  | 1-2 | 0.67 (0.60-0.74) | 0.75 (0.68-0.84) |
|  |  | WCC | >2 | reference | reference |
|  |  |  | 1-2 | 0.75 (0.65-0.87) | a |
|  |  | Duration of surgery | Q4 | reference | reference |
|  |  |  | Q1 | 0.54 (0.45-0.64) | 0.56 (0.46-0.68) |
|  |  |  | Q2 | 0.62 (0.54-0.70) | 0.69 (0.61-0.78) |
|  |  |  | Q3 | 0.76 (0.67-0.85) | 0.80 (0.71-0.89) |
|  |  | Season | Winter | reference | reference |
|  |  |  | Spring | a | a |
|  |  |  | Summer | a | a |
|  |  |  | Autumn | a | a |
|  | Appendectomy (endoscopic) | Age | <35 | reference | reference |
|  |  |  | 35-54 | a | a |
|  |  |  | 55-74 | a | a |
|  |  |  | ≥ 75 | a | a |
|  |  | ASA | >2 | reference | reference |
|  |  |  | 1-2 | a | a |
|  |  | WCC | >2 | reference | reference |
|  |  |  | 1-2 | a | a |
|  |  | Duration of surgery | Q4 | reference | reference |
|  |  |  | Q1 | 0.18 (0.06-0.49) | 0.29 (0.17-0.50) |
|  |  |  | Q2 | 0.39 (0.19-0.83) | 0.44 (0.25-0.78) |
|  |  |  | Q3 | 0.42 (0.27-0.66) | 0.43 (0.23-0.81) |
|  |  | Season | Winter | reference | reference |
|  |  |  | Spring | a | a |
|  |  |  | Summer | a | a |
|  |  |  | Autumn | a | a |
| **Heart and vascular surgery** | All | Age | <35 | reference | reference |
|  |  |  | 35-54 | 2.73 (1.29-5.78) | 1.62 (0.71-3.74) |
|  |  |  | 55-74 | 3.00 (1.33-6.73) | 2.00 (0.86-4.63) |
|  |  |  | ≥ 75 | 2.71 (1.18-6.24) | 2.21 (0.95-5.16) |
|  |  | ASA | >2 | reference | reference |
|  |  |  | 1-2 | 0.24 (0.17-0.33) | 0.55 (0.43-0.70) |
|  |  | WCC | >2 | reference | reference |
|  |  |  | 1-2 | 0.54 (0.36-0.81) | 0.42 (0.31-0.58) |
|  |  | Duration of surgery | Q4 | reference | reference |
|  |  |  | Q1 | 0.48 (0.39-0.61) | 0.56 (0.47-0.66) |
|  |  |  | Q2 | 0.68 (0.52-0.88) | 0.69 (0.61-0.79) |
|  |  |  | Q3 | 0.82 (0.70-0.96) | 0.83 (0.74-0.93) |
|  |  | Season | Winter | reference | reference |
|  |  |  | Spring | a | 0.97 (0.88-1.08) |
|  |  |  | Summer | a | 1.05 (0.94-1.18) |
|  |  |  | Autumn | a | 0.90 (0.82-0.98) |
|  | CABG (incl. vein harvesting) | Age | <35 | reference | reference |
|  |  |  | 35-54 | a | a |
|  |  |  | 55-74 | a | a |
|  |  |  | ≥ 75 | a | a |
|  |  | ASA | >2 | reference | reference |
|  |  |  | 1-2 | a | a |
|  |  | WCC | >2 | reference | reference |
|  |  |  | 1-2 | a | a |
|  |  | Duration of surgery | Q4 | reference | reference |
|  |  |  | Q1 | 0.55 (0.46-0.66) | 0.68 (0.58-0.81) |
|  |  |  | Q2 | 0.63 (0.51-0.79) | 0.82 (0.71-0.94) |
|  |  |  | Q3 | 0.83 (0.67-1.03) | 0.90 (0.79-1.02) |
|  |  | Season | Winter | reference | reference |
|  |  |  | Spring | a | a |
|  |  |  | Summer | a | a |
|  |  |  | Autumn | a | a |
|  | CABG (without vein harvesting) | Age | <35 | reference¹ | reference |
|  |  |  | 35-54 |  | a |
|  |  |  | 55-74 | 0.99 (0.75-1.31) | a |
|  |  |  | ≥ 75 | 0.70 (0.48-1.04) | a |
|  |  | ASA | >2 | reference | reference |
|  |  |  | 1-2 | a | a |
|  |  | WCC | >2 | reference | reference |
|  |  |  | 1-2 | b | b |
|  |  | Duration of surgery | Q4 | reference | reference |
|  |  |  | Q1 | a | 0.45 (0.32-0.62) |
|  |  |  | Q2 | a | 0.53 (0.40-0.71) |
|  |  |  | Q3 | a | 0.69 (0.51-0.93) |
|  |  | Season | Winter | reference | reference |
|  |  |  | Spring | a | a |
|  |  |  | Summer | a | a |
|  |  |  | Autumn | a | a |
|  | Re-vascularization of arterial occlusion | Age | <35 | reference | reference¹ |
|  |  |  | 35-54 | a |  |
|  |  |  | 55-74 | a | 1.45 (0.99-2.11) |
|  |  |  | ≥ 75 | a | 1.76 (1.19-2.61) |
|  |  | ASA | >2 | reference | reference |
|  |  |  | 1-2 | 0.61 (0.46-0.82) | 0.82 (0.70-0.98) |
|  |  | WCC | >2 | reference | reference |
|  |  |  | 1-2 | 0.40 (0.25-0.62) | 0.43 (0.30-0.62) |
|  |  | Duration of surgery | Q4 | reference | reference |
|  |  |  | Q1 | 0.38 (0.28-0.49) | 0.37 (0.29-0.45) |
|  |  |  | Q2 | 0.55 (0.41-0.74) | 0.52 (0.41-0.67) |
|  |  |  | Q3 | 0.80 (0.64-1.00) | 0.76 (0.62-0.92) |
|  |  | Season | Winter | reference | reference |
|  |  |  | Spring | a | a |
|  |  |  | Summer | a | a |
|  |  |  | Autumn | a | a |
|  | Venous stripping | Age | <35 | reference | reference |
|  |  |  | 35-54 | a | a |
|  |  |  | 55-74 | a | a |
|  |  |  | ≥ 75 | a | a |
|  |  | ASA | >2 | reference | reference |
|  |  |  | 1-2 | a | a |
|  |  | WCC | >2 | reference | reference |
|  |  |  | 1-2 | a | a |
|  |  | Duration of surgery | Q4 | reference | reference |
|  |  |  | Q1 | a | a |
|  |  |  | Q2 | a | a |
|  |  |  | Q3 | a | a |
|  |  | Season | Winter | reference | reference |
|  |  |  | Spring | a | a |
|  |  |  | Summer | a | a |
|  |  |  | Autumn | a | a |
| **Neurosurgery** | All | Age | <35 | reference | reference |
|  |  |  | 35-54 | 4.75 (0.83-27.31) | a |
|  |  |  | 55-74 | 4.99 (0.62-39.91) | a |
|  |  |  | ≥ 75 | 7.11 (0.99-50.89) | a |
|  |  | ASA | >2 | reference | reference |
|  |  |  | 1-2 | a | 0.39 (0.25-0.59) |
|  |  | WCC | >2 | reference | reference |
|  |  |  | 1-2 | a | a |
|  |  | Duration of surgery | Q4 | reference | reference |
|  |  |  | Q1 | 0.08 (0.03-0.21) | a |
|  |  |  | Q2 | 0.26 (0.12-0.55) | a |
|  |  |  | Q3 | 0.29 (0.12-0.68) | a |
|  |  | Season | Winter | reference | reference |
|  |  |  | Spring | 2.30 (1.06-4.98) | a |
|  |  |  | Summer | 1.37 (0.57-3.32) | a |
|  |  |  | Autumn | 3.04 (1.59-5.93) | a |
|  | Lumbar disk surgery | Age | <35 | reference | reference |
|  |  |  | 35-54 | 4.75 (0.83-27.31) | a |
|  |  |  | 55-74 | 4.99 (0.62-39.91) | a |
|  |  |  | ≥ 75 | 7.11 (0.99-50.89) | a |
|  |  | ASA | >2 | reference | reference |
|  |  |  | 1-2 | a | 0.39 (0.25-0.59) |
|  |  | WCC | >2 | reference | reference |
|  |  |  | 1-2 | a | b |
|  |  | Duration of surgery | Q4 | reference | reference |
|  |  |  | Q1 | 0.08 (0.03-0.21) | a |
|  |  |  | Q2 | 0.26 (0.12-0.55) | a |
|  |  |  | Q3 | 0.29 (0.12-0.68) | a |
|  |  | Season | Winter | reference | reference |
|  |  |  | Spring | 2.30 (1.06-4.98) | a |
|  |  |  | Summer | 1.37 (0.57-3.32) | a |
|  |  |  | Autumn | 3.04 (1.59-5.93) | a |
| **General surgery** | All | Age | <35 | reference | reference |
|  |  |  | 35-54 | 0.84 (0.33-2.16) | a |
|  |  |  | 55-74 | 1.63 (0.69-3.85) | a |
|  |  |  | ≥ 75 | 2.13 (0.87-5.23) | a |
|  |  | ASA | >2 | reference | reference |
|  |  |  | 1-2 | a | 0.46 (0.35-0.60) |
|  |  | WCC | >2 | reference | reference |
|  |  |  | 1-2 | a | 0.12 (0.05-0.29) |
|  |  | Duration of surgery | Q4 | reference | reference |
|  |  |  | Q1 | 0.35 (0.20-0.61) | 0.27 (0.14-0.50) |
|  |  |  | Q2 | 0.46 (0.27-0.77) | 0.68 (0.47-0.98) |
|  |  |  | Q3 | 0.50 (0.30-0.82) | 0.67 (0.46-0.98) |
|  |  | Season | Winter | reference | reference |
|  |  |  | Spring | a | a |
|  |  |  | Summer | a | a |
|  |  |  | Autumn | a | a |
|  | Hernia repair (endoscopic) | Age | <35 | reference | reference |
|  |  |  | 35-54 | a | a |
|  |  |  | 55-74 | a | a |
|  |  |  | ≥ 75 | a | a |
|  |  | ASA | >2 | reference | reference |
|  |  |  | 1-2 | a | a |
|  |  | WCC | >2 | reference | reference |
|  |  |  | 1-2 | b | a |
|  |  | Duration of surgery | Q4 | reference | reference |
|  |  |  | Q1 | a | 0.18 (0.04-0.74) |
|  |  |  | Q2 | a | 0.81 (0.37-1.79) |
|  |  |  | Q3 | a | 0.87 (0.40-1.91) |
|  |  | Season | Winter | reference | reference |
|  |  |  | Spring | a | a |
|  |  |  | Summer | a | a |
|  |  |  | Autumn | a | a |
|  | Hernia repair (open) | Age | <35 | reference | reference |
|  |  |  | 35-54 | a | a |
|  |  |  | 55-74 | a | a |
|  |  |  | ≥ 75 | a | a |
|  |  | ASA | >2 | reference | reference |
|  |  |  | 1-2 | a | 0.54 (0.37-0.79) |
|  |  | WCC | >2 | reference | reference |
|  |  |  | 1-2 | b | a |
|  |  | Duration of surgery | Q4 | reference | reference |
|  |  |  | Q1 | a | 0.30 (0.13-0.69) |
|  |  |  | Q2 | a | 0.64 (0.37-1.11) |
|  |  |  | Q3 | a | 0.59 (0.36-0.97) |
|  |  | Season | Winter | reference | reference |
|  |  |  | Spring | a | a |
|  |  |  | Summer | a | a |
|  |  |  | Autumn | a | a |
|  | Thyroid surgery | Age | <35 | reference | reference |
|  |  |  | 35-54 | 0.71 (0.23-2.21) | a |
|  |  |  | 55-74 | 1.35 (0.48-3.78) | a |
|  |  |  | ≥ 75 | 3.10 (1.06-9.08) | a |
|  |  | ASA | >2 | reference | reference |
|  |  |  | 1-2 | a | 0.27 (0.16-0.46) |
|  |  | WCC | >2 | reference | reference |
|  |  |  | 1-2 | b | a |
|  |  | Duration of surgery | Q4 | reference | reference |
|  |  |  | Q1 | a | a |
|  |  |  | Q2 | a | a |
|  |  |  | Q3 | a | a |
|  |  | Season | Winter | reference | reference |
|  |  |  | Spring | a | a |
|  |  |  | Summer | a | a |
|  |  |  | Autumn | a | a |

*OR* odds ratio; *CI* confidence interval; *ASA* American Society of Anesthesiologists; *WCC* wound contamination class; *Q1* first quartile; *Q2* second quartile; *Q3* third quartile; *Q4* forth quartile; *a* factor was eliminated if the p-value was greater than 0.05; *¹* groups were merged because no surgical site infections occurred in the original reference group

**Table c** Multivariable analysis of risk factors for the occurrence of deep and organ-space surgical site infections separated for female and male patients

| **Surgical category** | **Procedure** | **Variable** | **Group** | **OR women**  (95% CI) | **OR men**  (95% CI) |
| --- | --- | --- | --- | --- | --- |
| **All** |  | Age | <35 | reference | reference |
|  |  |  | 35-54 | 2.05 (1.61-2.61) | 1.59 (1.28-1.97) |
|  |  |  | 55-74 | 2.49 (1.95-3.18) | 2.14 (1.73-2.64) |
|  |  |  | ≥ 75 | 2.81 (2.17-3.64) | 2.42 (1.93-3.03) |
|  |  | ASA | >2 | reference | reference |
|  |  |  | 1-2 | 0.29 (0.25-0.33) | 0.37 (0.33-0.42) |
|  |  | WCC | >2 | reference | reference |
|  |  |  | 1-2 | 0.36 (0.30-0.43) | 0.39 (0.33-0.42) |
|  |  | Duration of surgery | Q4 | reference | reference |
|  |  |  | Q1 | 0.56 (0.49-0.64) | 0.57 (0.51-0.64) |
|  |  |  | Q2 | 0.63 (0.56-0.71) | 0.71 (0.64-0.78) |
|  |  |  | Q3 | 0.75 (0.69-0.82) | 0.82 (0.76-0.87) |
|  |  | Season | Winter | reference | reference |
|  |  |  | Spring | 1.08 (1.00-1.17) | 0.96 (0.92-1.08) |
|  |  |  | Summer | 1.24 (1.14-1.35) | 1.12 (1.03-1.21) |
|  |  |  | Autumn | 1.07 (0.98-1.15) | 1.02 (0.95-1.11) |
| **Orthopedics and traumatology** | **All** | Age | <35 | reference | reference |
|  |  |  | 35-54 | 3.63 (1.33-9.87) | 1.27 (0.79-2.03) |
|  |  |  | 55-74 | 3.87 (1.45-10.34) | 1.49 (0.92-2.41) |
|  |  |  | ≥ 75 | 6.01 (2.27-15.91) | 2.04 (1.25-3.13) |
|  |  | ASA | >2 | reference | reference |
|  |  |  | 1-2 | 0.38 (0.34-0.42) | 0.54 (0.47-0.61) |
|  |  | WCC | >2 | reference | reference |
|  |  |  | 1-2 | 0.27 (0.15-0.49) | 0.26 (0.15-0.45) |
|  |  | Duration of surgery | Q4 | reference | reference |
|  |  |  | Q1 | 0.47 (0.40-0.55) | 0.51 (0.43-0.60) |
|  |  |  | Q2 | 0.59 (0.52-0.67) | 0.64 (0.56-0.74) |
|  |  |  | Q3 | 0.71 (0.64-0.79) | 0.80 (0.71-0.90) |
|  |  | Season | Winter | reference | reference |
|  |  |  | Spring | 1.10 (0.98-1.24) | 1.04 (0.91-1.20) |
|  |  |  | Summer | 1.11 (0.99-1.26) | 1.14 (0.99-1.30) |
|  |  |  | Autumn | 1.32 (1.16-1.50) | 1.21 (1.06-1.37) |
|  | Hip prosthesis following arthrosis | Age | <35 | reference¹ | reference |
|  |  |  | 35-54 |  | 1.14 (0.43-3.00) |
|  |  |  | 55-74 | 1.43 (1.08-1.89) | 1.53 (0.58-4.04) |
|  |  |  | ≥ 75 | 1.66 (1.20-2.29) | 1.90 (0.72-5.00) |
|  |  | ASA | >2 | reference | reference |
|  |  |  | 1-2 | 0.38 (0.33-0.44) | 0.55 (0.47-0.64) |
|  |  | WCC | >2 | reference | reference |
|  |  |  | 1-2 | 0.26 (0.11-0.63) | 0.26 (0.14-0.48) |
|  |  | Duration of surgery | Q4 | reference | reference |
|  |  |  | Q1 | 0.41 (0.33-0.51) | 0.48 (0.38-0.60) |
|  |  |  | Q2 | 0.58 (0.49-0.69) | 0.66 (0.56-0.79) |
|  |  |  | Q3 | 0.65 (0.56-0.76) | 0.79 (0.67-0.93) |
|  |  | Season | Winter | reference | reference |
|  |  |  | Spring | 1.21 (1.01-1.45) | a |
|  |  |  | Summer | 1.40 (1.16-1.67) | a |
|  |  |  | Autumn | 1.15 (0.97-1.37) | a |
|  | Hip prosthesis following fracture | Age | <35 | reference¹ | reference¹ |
|  |  |  | 35-54 |  |  |
|  |  |  | 55-74 | a | a |
|  |  |  | ≥ 75 | a | a |
|  |  | ASA | >2 | reference | reference |
|  |  |  | 1-2 | 0.54 (0.45-0.65) | 0.73 (0.54-0.98) |
|  |  | WCC | >2 | reference | reference |
|  |  |  | 1-2 | a | a |
|  |  | Duration of surgery | Q4 | reference | reference |
|  |  |  | Q1 | 0.55 (0.44-0.70) | 0.60 (0.42-0.86) |
|  |  |  | Q2 | 0.65 (0.53-0.79) | 0.75 (0.55-1.03) |
|  |  |  | Q3 | 0.76 (0.63-0.92) | 1.00 (0.75-1.33) |
|  |  | Season | Winter | reference | reference |
|  |  |  | Spring | a | a |
|  |  |  | Summer | a | a |
|  |  |  | Autumn | a | a |
|  | Knee prosthesis | Age | <35 | reference¹ | reference¹ |
|  |  |  | 35-54 |  |  |
|  |  |  | 55-74 | a | 0.73 (0.53-1.01) |
|  |  |  | ≥ 75 | a | 1.03 (0.72-1.48) |
|  |  | ASA | >2 | reference | reference |
|  |  |  | 1-2 | 0.55 (0.43-0.71) | 0.63 (0.49-0.82) |
|  |  | WCC | >2 | reference | reference |
|  |  |  | 1-2 | 0.12 (0.05-0.26) | 0.14 (0.06-0.32) |
|  |  | Duration of surgery | Q4 | reference | reference |
|  |  |  | Q1 | 0.45 (0.34-0.61) | 0.57 (0.42-0.78) |
|  |  |  | Q2 | 0.55 (0.41-0.72) | 0.60 (0.47-0.77) |
|  |  |  | Q3 | 0.78 (0.59-1.03) | 0.72 (0.57-0.91) |
|  |  | Season | Winter | reference | reference |
|  |  |  | Spring | 1.47 (1.12-1.92) | 1.40 (1.04-1.87) |
|  |  |  | Summer | 1.75 (1.30-2.35) | 1.60 (1.23-2.09) |
|  |  |  | Autumn | 1.39 (0.99-1.95) | 1.51 (1.17-1.95) |
|  | Arthroscopic procedures | Age | <35 | reference | reference¹ |
|  |  |  | 35-54 | a |  |
|  |  |  | 55-74 | a | a |
|  |  |  | ≥ 75 | a | a |
|  |  | ASA | >2 | reference | reference |
|  |  |  | 1-2 | a | a |
|  |  | WCC | >2 | reference | reference |
|  |  |  | 1-2 | a | a |
|  |  | Duration of surgery | Q4 | reference | reference |
|  |  |  | Q1 | a | 0.11 (0.03-0.36) |
|  |  |  | Q2 | a | 0.23 (0.09-0.63) |
|  |  |  | Q3 | a | 0.62 (0.27-1.40) |
|  |  | Season | Winter | reference | reference |
|  |  |  | Spring | a | a |
|  |  |  | Summer | a | a |
|  |  |  | Autumn | a | a |
| **Abdominal surgery** | All | Age | <35 | reference | reference |
|  |  |  | 35-54 | 2.10 (1.61-2.75) | 1.72 (1.31-2.25) |
|  |  |  | 55-74 | 3.90 (3.00-5.08) | 3.04 (2.34-3.96) |
|  |  |  | ≥ 75 | 4.81 (3.53-6.56) | 3.33 (2.48-4.48) |
|  |  | ASA | >2 | reference | reference |
|  |  |  | 1-2 | 0.39 (0.33-0.45) | 0.45 (0.39-0.51) |
|  |  | WCC | >2 | reference | reference |
|  |  |  | 1-2 | 0.69 (0.57-0.85) | a |
|  |  | Duration of surgery | Q4 | reference | reference |
|  |  |  | Q1 | 0.62 (0.52-0.74) | 0.59 (0.49-0.71) |
|  |  |  | Q2 | 0.65 (0.56-0.75) | 0.73 (0.64-0.83) |
|  |  |  | Q3 | 0.75 (0.66-0.85) | 0.79 (0.70-0.89) |
|  |  | Season | Winter | reference | reference |
|  |  |  | Spring | a | a |
|  |  |  | Summer | a | a |
|  |  |  | Autumn | a | a |
|  | Cholecystectomy (endoscopic) | Age | <35 | reference | reference |
|  |  |  | 35-54 | 1.64 (0.74-3.63) | 0.51 (0.19-1.37) |
|  |  |  | 55-74 | 3.66 (1.60-7.08) | 2.68 (1.15-6.23) |
|  |  |  | ≥ 75 | 5.65 (2.95-10.83) | 4.46 (1.71-11.66) |
|  |  | ASA | >2 | reference | reference |
|  |  |  | 1-2 | 0.56 (0.40-0.78) | 0.62 (0.47-0.83) |
|  |  | WCC | >2 | reference | reference |
|  |  |  | 1-2 | 0.66 (0.49-0.88) | a |
|  |  | Duration of surgery | Q4 | reference | reference |
|  |  |  | Q1 | 0.53 (0.36-0.77) | 0.39 (0.25-0.62) |
|  |  |  | Q2 | 0.59 (0.41-0.84) | 0.61 (0.41-0.92) |
|  |  |  | Q3 | 0.69 (0.49-0.97) | 0.61 (0.41-0.90) |
|  |  | Season | Winter | reference | reference |
|  |  |  | Spring | a | a |
|  |  |  | Summer | a | a |
|  |  |  | Autumn | a | a |
|  | Colon surgery (endoscopic) | Age | <35 | reference | reference |
|  |  |  | 35-54 | a | a |
|  |  |  | 55-74 | a | a |
|  |  |  | ≥ 75 | a | a |
|  |  | ASA | >2 | reference | reference |
|  |  |  | 1-2 | 0.62 (0.48-0.81) | 0.61 (0.49-0.76) |
|  |  | WCC | >2 | reference | reference |
|  |  |  | 1-2 | a | a |
|  |  | Duration of surgery | Q4 | reference | reference |
|  |  |  | Q1 | 0.52 (0.37-0.75) | 0.47 (0.33-0.68) |
|  |  |  | Q2 | 0.57 (0.40-0.80) | 0.81 (0.60-1.10) |
|  |  |  | Q3 | 0.62 (0.44-0.88) | 0.82 (0.64-1.05) |
|  |  | Season | Winter | reference | reference |
|  |  |  | Spring | a | a |
|  |  |  | Summer | a | a |
|  |  |  | Autumn | a | a |
|  | Colon surgery (open) | Age | <35 | reference | reference |
|  |  |  | 35-54 | a | a |
|  |  |  | 55-74 | a | a |
|  |  |  | ≥ 75 | a | a |
|  |  | ASA | >2 | reference | reference |
|  |  |  | 1-2 | 0.63 (0.54-0.72) | 0.63 (0.54-0.73) |
|  |  | WCC | >2 | reference | reference |
|  |  |  | 1-2 | 0.75 (0.65-0.87) | a |
|  |  | Duration of surgery | Q4 | reference | reference |
|  |  |  | Q1 | 0.57 (0.47-0.69) | 0.53 (0.43-0.65) |
|  |  |  | Q2 | 0.62 (0.53-0.74) | 0.66 (0.57-0.76) |
|  |  |  | Q3 | 0.77 (0.66-0.90) | 0.76 (0.66-0.87) |
|  |  | Season | Winter | reference | reference |
|  |  |  | Spring | a | a |
|  |  |  | Summer | a | a |
|  |  |  | Autumn | a | a |
|  | Appendectomy (endoscopic) | Age | <35 | reference | reference |
|  |  |  | 35-54 | a | a |
|  |  |  | 55-74 | a | a |
|  |  |  | ≥ 75 | a | a |
|  |  | ASA | >2 | reference | reference |
|  |  |  | 1-2 | a | a |
|  |  | WCC | >2 | reference | reference |
|  |  |  | 1-2 | a | a |
|  |  | Duration of surgery | Q4 | reference | reference |
|  |  |  | Q1 | a | 0.17 (0.05-0.56) |
|  |  |  | Q2 | a | 0.29 (0.12-0.71) |
|  |  |  | Q3 | a | 0.52 (0.27-1.01) |
|  |  | Season | Winter | reference | Reference |
|  |  |  | Spring | a | a |
|  |  |  | Summer | a | a |
|  |  |  | Autumn | a | a |
| **Heart and vascular surgery** | All | Age | <35 | reference | reference |
|  |  |  | 35-54 | 3.59 (0.91-14.24) | 2.00 (0.69-5.80) |
|  |  |  | 55-74 | 3.99 (0.92-17.28) | 2.56 (0.89-7.34) |
|  |  |  | ≥ 75 | 3.56 (0.80-15.92) | 3.00 (1.04-8.65) |
|  |  | ASA | >2 | reference | reference |
|  |  |  | 1-2 | 0.16 (0.09-0.28) | 0.50 (0.36-0.69) |
|  |  | WCC | >2 | reference | reference |
|  |  |  | 1-2 | 0.45 (0.29-0.71) | 0.53 (0.34-0.81) |
|  |  | Duration of surgery | Q4 | reference | reference |
|  |  |  | Q1 | 0.50 (0.38-0.67) | 0.60 (0.49-0.74) |
|  |  |  | Q2 | 0.63 (0.45-0.88) | 0.75 (0.63-0.89) |
|  |  |  | Q3 | 0.78 (0.64-0.95) | 0.86 (0.74-1.01) |
|  |  | Season | Winter | reference | reference |
|  |  |  | Spring | a | a |
|  |  |  | Summer | a | a |
|  |  |  | Autumn | a | a |
|  | CABG (incl. vein harvesting) | Age | <35 | reference | reference |
|  |  |  | 35-54 | a | 0.27 (0.09-0.83) |
|  |  |  | 55-74 | a | 0.33 (0.11-0.97) |
|  |  |  | ≥ 75 | a | 0.38 (0.13-1.10) |
|  |  | ASA | >2 | reference | reference |
|  |  |  | 1-2 | a | 0.64 (0.47-0.88) |
|  |  | WCC | >2 | reference | reference |
|  |  |  | 1-2 | a | a |
|  |  | Duration of surgery | Q4 | reference | reference |
|  |  |  | Q1 | 0.60 (0.47-0.77) | 0.76 (0.62-0.93) |
|  |  |  | Q2 | 0.60 (0.45-0.80) | 0.93 (0.79-1.09) |
|  |  |  | Q3 | 0.82 (0.63-1.07) | 1.02 (0.87-1.20) |
|  |  | Season | Winter | reference | reference |
|  |  |  | Spring | a | a |
|  |  |  | Summer | a | a |
|  |  |  | Autumn | a | a |
|  | CABG (without vein harvesting) | Age | <35 | reference¹ | reference |
|  |  |  | 35-54 |  | a |
|  |  |  | 55-74 | a | a |
|  |  |  | ≥ 75 | a | a |
|  |  | ASA | >2 | reference | reference |
|  |  |  | 1-2 | a | a |
|  |  | WCC | >2 | reference | reference |
|  |  |  | 1-2 | b | b |
|  |  | Duration of surgery | Q4 | reference | reference |
|  |  |  | Q1 | a | 0.47 (0.31-0.70) |
|  |  |  | Q2 | a | 0.44 (0.31-0.63) |
|  |  |  | Q3 | a | 0.68 (0.45-1.02) |
|  |  | Season | Winter | reference | reference |
|  |  |  | Spring | a | a |
|  |  |  | Summer | a | a |
|  |  |  | Autumn | a | a |
|  | Re-vascularization of arterial occlusion | Age | <35 | reference | reference¹ |
|  |  |  | 35-54 | a |  |
|  |  |  | 55-74 | a | 1.18 (0.72-1.73) |
|  |  |  | ≥ 75 | a | 1.53 (0.97-2.40) |
|  |  | ASA | >2 | reference | reference |
|  |  |  | 1-2 | 0.60 (0.42-0.84) | a |
|  |  | WCC | >2 | reference | reference |
|  |  |  | 1-2 | 0.27 (0.17-0.42) | 0.45 (0.29-0.71) |
|  |  | Duration of surgery | Q4 | reference | reference |
|  |  |  | Q1 | 0.31 (0.18-0.54) | 0.32 (0.23-0.45) |
|  |  |  | Q2 | 0.53 (0.35-0.79) | 0.51 (0.36-0.72) |
|  |  |  | Q3 | 0.78 (0.57-1.07) | 0.63 (0.47-0.85) |
|  |  | Season | Winter | reference | reference |
|  |  |  | Spring | a | a |
|  |  |  | Summer | a | a |
|  |  |  | Autumn | a | a |
|  | Venous stripping | Age | <35 | reference | reference |
|  |  |  | 35-54 | a | a |
|  |  |  | 55-74 | a | a |
|  |  |  | ≥ 75 | a | a |
|  |  | ASA | >2 | reference | reference |
|  |  |  | 1-2 | a | a |
|  |  | WCC | >2 | reference | reference |
|  |  |  | 1-2 | a | a |
|  |  | Duration of surgery | Q4 | reference | reference |
|  |  |  | Q1 | a | a |
|  |  |  | Q2 | a | a |
|  |  |  | Q3 | a | a |
|  |  | Season | Winter | reference | reference |
|  |  |  | Spring | a | a |
|  |  |  | Summer | a | a |
|  |  |  | Autumn | a | a |
| **Neurosurgery** | All | Age | <35 | reference | reference |
|  |  |  | 35-54 | a | a |
|  |  |  | 55-74 | a | a |
|  |  |  | ≥ 75 | a | a |
|  |  | ASA | >2 | reference | reference |
|  |  |  | 1-2 | a | a |
|  |  | WCC | >2 | reference | reference |
|  |  |  | 1-2 | a | b |
|  |  | Duration of surgery | Q4 | reference | reference |
|  |  |  | Q1 | 0.09 (0.03-0.33) | a |
|  |  |  | Q2 | 0.34 (0.15-0.76) | a |
|  |  |  | Q3 | 0.24 (0.08-0.77) | a |
|  |  | Season | Winter | reference | reference |
|  |  |  | Spring | a | a |
|  |  |  | Summer | a | a |
|  |  |  | Autumn | a | a |
|  | Lumbar disk surgery | Age | <35 | reference | reference |
|  |  |  | 35-54 | a | a |
|  |  |  | 55-74 | a | a |
|  |  |  | ≥ 75 | a | a |
|  |  | ASA | >2 | reference | reference |
|  |  |  | 1-2 | a | a |
|  |  | WCC | >2 | reference | reference |
|  |  |  | 1-2 | a | b |
|  |  | Duration of surgery | Q4 | reference | reference |
|  |  |  | Q1 | 0.09 (0.03-0.33) | a |
|  |  |  | Q2 | 0.34 (0.15-0.76) | a |
|  |  |  | Q3 | 0.24 (0.08-0.77) | a |
|  |  | Season | Winter | reference | reference |
|  |  |  | Spring | a | a |
|  |  |  | Summer | a | a |
|  |  |  | Autumn | a | a |
| **General surgery** | All | Age | <35 | reference | reference |
|  |  |  | 35-54 | a | a |
|  |  |  | 55-74 | a | a |
|  |  |  | ≥ 75 | a | a |
|  |  | ASA | >2 | reference | reference |
|  |  |  | 1-2 | 0.25 (0.14-0.47) | 0.39 (0.25-0.60) |
|  |  | WCC | >2 | reference | reference |
|  |  |  | 1-2 | a | 0.44 (0.02-0.11) |
|  |  | Duration of surgery | Q4 | reference | reference |
|  |  |  | Q1 | a | 0.25 (0.08-0.77) |
|  |  |  | Q2 | a | 0.74 (0.39-1.41) |
|  |  |  | Q3 | a | 0.53 (0.28-1.00) |
|  |  | Season | Winter | reference | reference |
|  |  |  | Spring | a | a |
|  |  |  | Summer | a | a |
|  |  |  | Autumn | a | a |
|  | Hernia repair (endoscopic) | Age | <35 | reference | reference |
|  |  |  | 35-54 | b | a |
|  |  |  | 55-74 | b | a |
|  |  |  | ≥ 75 | b | a |
|  |  | ASA | >2 | reference | reference |
|  |  |  | 1-2 | b | a |
|  |  | WCC | >2 | reference | reference |
|  |  |  | 1-2 | b | a |
|  |  | Duration of surgery | Q4 | reference | reference |
|  |  |  | Q1 | b | a |
|  |  |  | Q2 | b | a |
|  |  |  | Q3 | b | a |
|  |  | Season | Winter | reference | reference |
|  |  |  | Spring | b | a |
|  |  |  | Summer | b | a |
|  |  |  | Autumn | b | a |
|  | Hernia repair (open) | Age | <35 | reference | reference |
|  |  |  | 35-54 | a | a |
|  |  |  | 55-74 | a | a |
|  |  |  | ≥ 75 | a | a |
|  |  | ASA | >2 | reference | reference |
|  |  |  | 1-2 | a | 0.45 (0.26-0.78) |
|  |  | WCC | >2 | reference | reference |
|  |  |  | 1-2 | b | a |
|  |  | Duration of surgery | Q4 | reference | reference |
|  |  |  | Q1 | b | a |
|  |  |  | Q2 | b | a |
|  |  |  | Q3 | b | a |
|  |  | Season | Winter | reference | reference |
|  |  |  | Spring | a | a |
|  |  |  | Summer | a | a |
|  |  |  | Autumn | a | a |
|  | Thyroid surgery | Age | <35 | reference | reference |
|  |  |  | 35-54 | a | a |
|  |  |  | 55-74 | a | a |
|  |  |  | ≥ 75 | a | a |
|  |  | ASA | >2 | reference | reference |
|  |  |  | 1-2 | 0.24 (0.12-0.48) | 0.22 (0.10-0.48) |
|  |  | WCC | >2 | reference | reference |
|  |  |  | 1-2 | a | a |
|  |  | Duration of surgery | Q4 | reference | reference |
|  |  |  | Q1 | a | a |
|  |  |  | Q2 | a | a |
|  |  |  | Q3 | a | a |
|  |  | Season | Winter | reference | reference |
|  |  |  | Spring | a | a |
|  |  |  | Summer | a | a |
|  |  |  | Autumn | a | a |

*OR* odds ratio; *CI* confidence interval; *ASA* American Society of Anesthesiologists; *WCC* wound contamination class; *Q1* first quartile; *Q2* second quartile; *Q3* third quartile; *Q4* forth quartile; *a* factor was eliminated if the p-value was greater than 0.05; *b* factor was not included because no surgical site infections occurred in at least one of the factor levels, thus the effect of the factor could not be estimated; *¹* groups were merged because no surgical site infections occurred in the original reference group
